# Toward a General-Purpose Heterogeneous Ensemble for Pattern Classification

**DOI:** 10.1155/2015/909123

**Published:** 2015-08-27

**Authors:** Loris Nanni, Sheryl Brahnam, Stefano Ghidoni, Alessandra Lumini

**Affiliations:** ^1^DEI, University of Padova, Via Gradenigo 6, 35131 Padova, Italy; ^2^Computer Information Systems, Missouri State University, 901 S. National, Springfield, MO 65804, USA; ^3^DISI, Università di Bologna, Via Sacchi 3, 47521 Cesena, Italy

## Abstract

We perform an extensive study of the performance of different classification approaches on twenty-five datasets (fourteen image datasets and eleven UCI data mining datasets). The aim is to find General-Purpose (GP) heterogeneous ensembles (requiring little to no parameter tuning) that perform competitively across multiple datasets. The state-of-the-art classifiers examined in this study include the support vector machine, Gaussian process classifiers, random subspace of adaboost, random subspace of rotation boosting, and deep learning classifiers. We demonstrate that a heterogeneous ensemble based on the simple fusion by sum rule of different classifiers performs consistently well across all twenty-five datasets. The most important result of our investigation is demonstrating that some very recent approaches, including the heterogeneous ensemble we propose in this paper, are capable of outperforming an SVM classifier (implemented with LibSVM), even when both kernel selection and SVM parameters are carefully tuned for each dataset.

## 1. Introduction

The present trend in machine learning is focused on building optimal classification systems for very specific, well-defined problems. Another research focus, however, would be to work on building General-Purpose (GP) systems that are capable of handling a broader range of problems as well as multiple data types. Ideally, GP systems would work well out of the box, requiring little to no parameter tuning but would still perform competitively against less flexible systems that have been optimized for very specific problems and datasets. One promising avenue of exploration is to build ensembles that are composed of diverse classifiers that merge their hypotheses [1], thereby resulting in a better approximation of a true hypothesis [2].

Many ensemble construction techniques are available. One approach is to perturb the information that is given to the base classifiers. The basic assumption behind this approach is that each of the base classifiers makes errors that are independent of each other, but as part of an ensemble they offer stronger classificatory power. To build an ensemble using this approach, *K* training sets are first created, and *K* classifiers are then trained on each of the *K* training sets. The results of the *K* classifiers are combined using some decision rule such as majority voting, sum rule, max rule, min rule, product rule, median rule, and Borda count. Different types of perturbations methods have been developed to maximize the classifier diversity in an ensemble. These methods focus on perturbing the training patterns, the feature sets, the classifiers, or some combination of these perturbation methods.

In pattern perturbation, *K* new training sets are created (commonly following an iterative approach) by perturbing the original training set, and a different classifier is trained on each new set. Some well-known pattern perturbation techniques include Bagging [3], Arcing [4], Class Switching [5], and Decorate [6]. In Bagging [3], new training sets are subsets of the original training data. In Arcing [4], each new training set is created based on the misclassified patterns in the previous iteration. In Class Switching [5], new training sets are created by randomly changing the labels of a subset of the original training data. Decorate [6] creates new training sets by adding artificial patterns misclassified by the combined decision of the ensemble.

Feature perturbation techniques manipulate a set of original features into new training sets composed of perturbed features. Some important examples of feature perturbation include random subspace (RS) [7] and Input Decimated Ensemble [8]. In RS [7], *K* new training sets are randomly generated from subsets of the feature set. In Input Decimated Ensemble [8], new training sets are generated using the principal component analysis (PCA) transform, where PCA is calculated on the training patterns belonging to each particular class. The ensemble size is thus bounded by the number of classes. This limitation can be avoided, however, as shown in [9], if PCA is performed on training patterns that have been partitioned into clusters.

New ensembles can also be composed by mixing the two perturbation methods discussed above. For example, Random Forest [10] uses a bagging ensemble of decision trees, where a random selection of features is used to split a given node.

Finally, in classifier perturbation, each classifier of the same type (homogeneous ensembles) can be given different parameter values, or different classifiers (heterogeneous ensembles) can be combined and trained on the same training set. Classifier perturbation methods for building ensembles have been the least studied in the literature, but recently several studies have focused on this type of ensemble [11]. Moreover, several papers have investigated building GP heterogeneous ensembles [12, 13]. In [12], an ensemble combining the RS approach with an ensemble using an editing approach to reduce outliers was compared with other state-of-the-art methods across sixteen benchmark datasets representing very different problems (numerous medical problems, image problems, a vowel dataset, a credit dataset, etc.). Although none of the ensembles investigated in [12] worked consistently well across all sixteen datasets, one GP ensemble worked well across all the image datasets. Moreover, in some cases, the GP ensemble performed better than an SVM whose parameters had been optimally tuned on a specific dataset.

GP ensembles that exploit information available in different feature extraction methods and representations of the data have also been explored. In [13], for instance, the goal was to search for a GP ensemble for protein classification that combined an optimal set of different protein representations and descriptors and that performed well across fourteen protein classification datasets representing different protein classification tasks. It was discovered in [13] that large descriptors work better when a large training set is available (due to the curse of dimensionality). Although no ensemble was discovered that provided the best performance across all fourteen datasets, it was shown that it is always possible to find a more limited GP ensemble that performed well across each type of dataset.

In this work the focus is on testing different classifiers and their combinations across twenty-five datasets (fourteen image datasets and eleven UCI data mining datasets). In the image datasets, two state-of-the-art texture descriptors are utilized: Local Ternary Patterns [14] and Local Phase Quantization [15]. As the majority of machine learning papers published in the literature are based on the LibSVM implementation of SVM, the aim of this work is to compare the performance of the LibSVM library with several recently proposed classifiers (Gaussian process classifiers, RS of AdaBoost, RS of rotation boosting, and deep learning) and to show that a heterogeneous GP ensemble of classifiers works well across the different datasets. For all the classifiers compared in this study, we use well-known toolboxes that have been extensively tested and that are freely available. Moreover, to make results reproducible and to gain a wider diffusion of this type of research, the MATLAB code/interface for building the GP heterogeneous ensembles proposed in this work is provided. We hope this tool will also prove useful for practitioners.

The most interesting result obtained from our experiments is that the best GP ensemble proposed in this paper outperforms each stand-alone classifier without any ad hoc tuning on the dataset: the same fusion rule is used for all twenty-five datasets tested in this work. As a result, we are confident that the proposed GP ensemble can easily be extended to other problems and should prove useful to researchers who want a reliable classifier that works well without tuning it. It should be noted, however, that there is a cost associated with using heterogeneous ensembles: increased computational time.

The remainder of this paper is organized as follows. In Section 2, the different classifiers explored in this paper are briefly described. In Section 3, the feature descriptors are outlined. In Section 4, we provide an overview of the twenty-five datasets. In Section 5, we present the experimental results along with our best GP heterogeneous ensembles. We conclude in Section 6 with a few reflections and remarks on some issues involved in developing GP ensembles and list some future directions of research. The MATLAB code for all the classifiers used in the proposed ensembles are available at https://www.dei.unipd.it/node/2357. Moreover, for the purpose of reproducing and comparing results, the split training/testing sets are also available at the above website.

## 2. Classifiers

Since the aim of this study is to find a heterogeneous multiclassifier system that works well with a large number of datasets, we examined the fusion by sum rule of several state-of-the-art classifiers: the Support Vector Machine (SVM), Gaussian process classifier (GPC), RS of AdaBoost (RS_AB), RS of rotation boosting (RS_RB), and deep learning (DL). The sum rule [2] simply sums the matching scores (normalized to mean 0 and standard deviation 1) provided by each of the different classifier systems. Each of the classifiers examined in this study is described briefly below.

### 2.1. Support Vector Machine (SVM)

SVM [16] is a binary classifier and is used as the core classifiers in several of our ensembles. An SVM performs classification by cutting the *n*-dimensional space (*n* being the number of features) into two regions associated with two distinct classes, often referred to as the* positive class* and the* negative class*. The regions are separated by an *n*-dimensional hyperplane that has the largest possible distance *d* from the training vectors of the two classes. Three kernels are tested in our experiments: linear, radial basis function, and polynomial. For each kernel, a dataset driven fine-tuning of parameters is performed. SVM is implemented using LibSVM, available at http://www.csie.ntu.edu.tw/~cjlin/libsvm/.

In addition to SVM implemented with LibSVM, we test an RS ensemble with SVM as the classifier (using fifty different subspaces that include 50% of the original features). This ensemble is called RS_SVM.

### 2.2. The Gaussian Process Classifier (GPC)

GPC [17] is a probabilistic approach for learning in kernel machines. It considers two procedures for approximating inference for binary classification: (1) Laplace's approximation, which is based on an expansion around the mode of the posterior, and (2) the Expectation Propagation algorithm, which is based on matching moments approximations to the marginals of the posterior. GPC is implemented using the MATLAB code available at http://www.gaussianprocess.org/gpml/code/matlab/doc/index.html.

### 2.3. Random Subspace of AdaBoost (RS_AB)

RS_AB [18] is a supervised learning algorithm that boosts the classification performance of a simple binary classifier by combining a collection of weak classifiers. The output of the weak learners is combined into a weighted sum that represents the final output of the boosted classifier. In this work, we combine AdaBoost with the RS method [7] for building ensembles. This results in a pseudorandom selection of subsets of components in the feature vector that are then used for training the different classifiers of the ensemble. We use RS to construct fifty different subspaces that include 50% of the original features. A different AdaBoost.M2 [18] is trained on each subspace. AdaBoost.M2 gives the weak learner (a neural network in our studies) more expressive power. The 50 classifiers are combined by sum rule.

### 2.4. Random Subspace of Rotation Boosting (RS_RB)

RS_RB is the random subspace version of rotation boosting (RB). RB [19] is an ensemble of decision trees based on randomly splitting the feature set into subsets. In each subset a feature transform is applied (PCA without feature reduction in the original version of RB). Instead of PCA, the feature transform used in this study is Neighborhood Preserving Embedding (NPE) as in [9]. NPE is described in Section 3.3.

### 2.5. Deep Learning (DL)

Deep learning is a recent and one of the best-performing approaches to Artificial Intelligence, a field that was revolutionized when it was first proposed in 2006 [20]. The main feature of deep learning is its layered structure: there are several layers of processing nodes between its input and output, with every layer adding a certain level of abstraction to the overall representation. For example, image interpretation is a task that can be performed through several steps: at a lower level, small image patches are considered, leading to features like edges and texture. Such low-level descriptors can be combined together to build a more complex representation: at the second level, for example, features like larger image patches and contours can be considered. Moving toward upper levels, the elements considered by the network are of increased complexity and are extracted from larger areas in the image (i.e., larger sets of input data).

Another major feature of deep learning networks is that they are able to exploit unlabeled data, a crucial feature when dealing with huge sets of data. Deep learning has been widely used in computer vision and image understanding applications, including object recognition in 3D (RGB-D) data [21] and face detection and verification [22].

In this work, we test the deep learning approach based on the Feedforward Backpropagation Neural Network (FBNN) [23] with a sigmoid activation function. We train FBNN for 10000 epochs with minibatches of size 25. Three versions of DL are tested DL1, DL2, and DL3. Each has an input layer that is the size of the input feature vector and an output layer that is the size of the number of classes. DL1 has a hidden layer of size 100. DL2 has two hidden layers each of size 100, and DL3 has two hidden layers each of size 500. DL is implemented using the MATLAB code available at http://it.mathworks.com/matlabcentral/fileexchange/38310-deep-learning-toolbox.

## 3. Feature Extraction

In Sections 3.1 and 3.2, we briefly describe the texture features used with the fourteen image datasets. Many methods are available for extracting features from texture. Two of the best-performing methods are Local Ternary Patterns (LTP) and Local Phase Quantization (LPQ). In Section 3.3, we describe the NPE descriptor that is used as a transform in RS_RB.

### 3.1. Local Ternary Patterns (LTP)

LTP [14] is an extension of the canonical Local Binary Pattern (LBP) operator designed to be more discriminant and less sensitive to noise in uniform regions. The LBP operator [24] is computed at each pixel *I*_*c*_ of an image by considering the differences between grey-level values of a small circular neighborhood (with radius *R* pixels):

(1) $$\begin{array}{c} \text{LBP}\left(P,R\right)=\overset{P-1}{\underset{i=0}{\sum }}s\left(I_{p}-I_{c}\right)2^{i}, \end{array}$$

where *P* is the number of pixels in the circular neighborhood *I*_*p*_ and *s*(*x*) is a threshold function such that

(2) $$\begin{array}{c} s\left(x\right)=\left\{\begin{array}{cc} 1, & x\geq 0\\ 0, & x<0. \end{array}\right. \end{array}$$

In the LTP, the threshold function *s*(*x*) is substituted by a ternary coding function that makes the operator more robust to noise.

The ternary coding *t*(*x*) is defined as

(3) $$\begin{array}{c} t\left(x\right)=\left\{\begin{array}{cc} 1, & x\geq \tau \\ 0, & \left|x\right|\leq \tau \\ -1, & x\leq -\tau , \end{array}\right. \end{array}$$

where *τ* is a threshold fixed to 3 in this work.

The ternary code obtained by the *t*(*x*) function is split into two binary codes by considering its positive and negative components, according to the following binary function *b*_*v*_(*t*(*x*)):

(4) $$\begin{array}{c} b_{v}\left(t\left(x\right)\right)=\left\{\begin{array}{cc} 1 & t\left(x\right)=v\\ 0 & \text{otherwise} \end{array}\right.\;v\in \left\{-1,1\right\}. \end{array}$$

Thus, the LTP_*v*_ operator for *v* ∈ { − 1, 1} is

(5) $$\begin{array}{c} {\text{LTP}}_{v}\left(P,R\right)=\overset{P-1}{\underset{p=0}{\sum }}b_{v}\left(t\left(I_{p}-I_{c}\right)\right)2^{n}. \end{array}$$

The resulting binary codes are used to create two histograms of LTP values.

In this work two values of *R* and *P* are used: (*R* = 1; *P* = 16) and (*R* = 2, *P* = 16); hence, we have four codes (two sets of parameters, with a positive and a negative code for each of them).

### 3.2. Local Phase Quantization (LPQ)

LPQ is a texture descriptor [15] based on the blur invariance of the Fourier Transform Phase. For each pixel position **x** of the image *f*(**x**), the 2D short-term Fourier transform (STFT) is computed over a rectangular neighborhood of size *L* × *L*, and four complex coefficients, corresponding to the 2D frequencies, are considered and quantized to construct the final descriptor: *u*_1_ = [*a*, 0]^*T*^, *u*_2_ = [0, *a*]^*T*^, *u*_3_ = [*a*, *a*]^*T*^, and *u*_4_ = [*a*, − *a*]^*T*^, where *a* is a scalar frequency parameter.

The four complex coefficients [*u*_1_, *u*_2_, *u*_3_, *u*_4_] need to be decorrelated before quantization to become statistically independent and maximally preserve the information. Assuming a Gaussian distribution and a fixed correlation coefficient between adjacent pixel values *ρ*, a whitening transform can be obtained from the singular value decomposition of the covariance matrix of the transform coefficient vector. After decorrelation, the vector *G*_*c*_ ∈ *ℜ* ^8^ that contains the decorrelated STFT coefficients for the pixel *I*_*c*_ is quantized using a scalar quantizer *s*(*x*) (already defined in (2)). Then the final LPQ code is represented as an integer between 0 and 255 using the binary coding:

(6) $$\begin{array}{c} \text{LPQ}\left(L\right)=\overset{8}{\underset{i=1}{\sum }}s\left(G_{c}\right)2^{i-1}. \end{array}$$

Finally, a histogram of these integer values is composed and used as a feature vector. In this work, we tested LPQ using two sizes for the local window *L* (3 and 5), both with Gaussian derivative quadrature filter pairs for local frequency estimation. LPQ is implemented using the MATLAB code available at http://www.cse.oulu.fi/CMV/Downloads/LPQMatlab.

### 3.3. Neighborhood Preserving Embedding (NPE)

First, proposed in [25], the NPE transformation is a global approach that preserves the local neighborhood structure on the data manifold. PCA, in contrast, preserves the global Euclidean structure. Thus, NPE is less sensitive to outliers than PCA. As described in Section 2, we use NPE as the transform for dimensionality reduction in RB.

Given a set of points *x*_1_, *x*_2_,…, *x*_*m*_ ∈ *R* ^*n*^, the idea behind NPE is to find a transformation matrix **A** that maps these points into another set *y*_1_, *y*_2_,…, *y*_*m*_ ∈ *R* ^*d*^, where *d* ≪ *n*.  In this way, *y*_*i*_ = *A* ^*T*^ *x*_*i*_ represents *x*_*i*_ in a space with significantly less dimensions.

NPE begins by building a weight matrix to describe the relationships between data points: each point is described as a weighted combination of its neighbors. An optimal embedding is sought such that the neighborhood structure is preserved in the reduced space.

The algorithm can be formalized in three steps:

(1) Build an adjacency graph: define a graph **G** with *m* nodes. The *i*th node represents the point *x*_*i*_. There is an edge between *i* and *j* if and only if *x*_*j*_ is one of the *K* nearest neighbors of *x*_*i*_.
(2) Compute weights: in this step weights on edges are calculated. **W** is the weight matrix and *w*_*ij*_ is the weight of the edge from node *i* to node *j*. The matrix can be computed by minimizing the objective function:
  (7) $$\begin{array}{c} \min\;\overset{m}{\underset{i}{\sum }}{\left\|x_{i}-\overset{m}{\underset{j}{\sum }}w_{ij}x_{j}\right\|}^{2}\\ \text{Subject to:}\;\overset{m}{\underset{j}{\sum }}w_{ij}=1,\;j=1,2,\ldots ,m. \end{array}$$
(3) Compute the projection: in this step the linear projection is computed. The following eigenvector problem is solved: *XMX* ^*T*^ **a** = ***λ*** *XX* ^*T*^ **a**; *M* = (*I* − *W*)′(*I* − *W*). The local manifold structure is then preserved using the following transformation matrix **A** that maps  *x*_*i*_ to *y*_*i*_:
  (8) $$\begin{array}{c} y_{i}=A^{T}x_{i},\;\text{where }A=\left(a_{0},a_{1},\ldots ,a_{d-1}\right). \end{array}$$

## 4. Datasets

To assess their generalizability, the approaches proposed in this paper were tested across twenty-five datasets: fourteen image classification datasets and eleven UCI data mining datasets.

### 4.1. Image Classification Datasets

The following fourteen image classification datasets that represent very different computer vision problems were selected to evaluate the generalizability of our approach:

- PS: this Pap Smear dataset [26] contains 917 images representing cells that are used in the diagnosis of cervical cancer.
- VI: this dataset, reported in [27], contains images of viruses. A split training/testing set is provided by the authors and is used in this paper. The masks for subtracting image backgrounds were not utilized.
- CH: this dataset, reported in [28], contains fluorescent microscopy images taken from Chinese hamster ovary cells that belong to five different classes.
- SM: this dataset, reported in [29], contains images extracted from video-based smoke detection surveillance systems. The same division of the dataset into training/testing sets, reported in [29], is used in this paper.
- HI: this dataset, reported in [30], contains images extracted from four fundamental tissues.
- BR: this dataset, reported in [31], contains 273 malignant and 311 benign breast cancer images.
- PR: this is a dataset containing 118 DNA-binding proteins and 231 non-DNA-binding proteins. Texture descriptors are extracted from the 2D distance matrix that represents each protein. This matrix is obtained from the 3D tertiary structure of a given protein considering only atoms that belong to the protein backbone (see [32] for details).
- HE: the 2D HeLa dataset [28] contains single cell images divided into 10 staining classes that were taken from fluorescence microscope acquisitions on HeLa cells.
- LO: the locate endogenous mouse subcellular organelles dataset [33] contains 502 images unevenly distributed among 10 classes of endogenous proteins or features of specific organelles.
- TR: the locate transfected mouse subcellular organelles dataset [33] contains 553 images unevenly distributed in 11 classes of fluorescence-tagged or epitope-tagged proteins transiently expressed in specific organelles.
- PI: this dataset, reported in [34], contains pictures extracted from digitalized pages of the Holy Bible of Borso d'Este, duke of Ferrara, Italy, from 1450 AD to 1471 AD. PI is composed of 13 classes, characterized by a clear semantic meaning and significant search relevance.
- RN: this is a dataset containing 200 fluorescence microscopy images evenly distributed among 10 classes of fly cells subjected to a set of gene knockdowns using RNAi. The cells were stained with DAPI to visualize their nuclei.
- PA: this dataset, reported in [35], contains 2338 paintings by 50 painters, representative of 13 different painting styles: abstract expressionism, baroque, constructivism, cubism, impressionism, neoclassical, pop art, postimpressionism, realism, renaissance, romanticism, surrealism, and symbolism. A split training/testing set is provided by the authors [35] and is used in this paper.
- LE: this dataset contains images of 20 species of Brazilian flora [36]. A total of 400 samples, divided into 20 classes (20 samples per class), were collected. Three windows were extracted from each sample. A constraint to the fivefold cross-validation technique was added that required that all windows extracted from a given leaf belong either to the training set or to the testing set, not both.

A descriptive summary of each dataset, along with the URL where each dataset can be downloaded, is reported in Table 1. If a dataset contains RGB images, these were converted to grey-level images before the feature extraction step. The testing protocol used with these datasets is the fivefold cross-validation method, with the exception of three dataset, SM, VI, and PA, where the protocols and testing/training sets defined by the datasets were used (these protocols, which are briefly described above, were obtained from the creators of each of these datasets).

### 4.2. UCI Data Mining Datasets

We report results obtained using eleven datasets from the UCI repository [37]. In Table 2 we list each dataset used in this study and describe each of them according to the number of attributes (#A), the number of samples (#S), and the number of classes (#C) that each contains. The testing protocol is the fivefold cross-validation method. All features in these datasets were linearly normalized between 0 and 1 before classification, using only the training data for normalizing the test data. In all the tests the testing set is completely blind.

## 5. Results and Discussion

The performance indicator used in all experiments is the area under the ROC curve (AUC) because it provides a better overview of classification results [38]. In the multiclass problem, AUC is calculated using the one-versus-all approach, where a given class is considered “positive” and all other classes are considered “negative.” The average AUC is reported in all tables.

In order to better compare the ensembles, we also consider their average AUC (Av) and the average rank (RA) obtained in all datasets. RA should be minimized: if a classifier obtains the perfect classification in all the datasets, its RA is 1. The last row labelled Av in all the tables included in this section reports the average AUC performance on all the datasets. To statistically validate these experiments, the Wilcoxon Signed-Rank test [39] was used for all methods.

For the purpose of reproducing and comparing results, the split training/testing sets used for each dataset are available at the website listed at the end of the Introduction. For the image datasets, we also provide both the LTP and LPQ features that were extracted from each dataset for this study.

### 5.1. Experimental Results in Image Classification

The first set of experiments are aimed at comparing the performance of the proposed approaches with the stand-alone methods described in Section 2. Tables 3 and 4 report the results obtained by the state-of-the-art classifiers, trained with LTP and LPQ, respectively, and the following heterogeneous ensembles based on the sum rule:

- S_D: (DL1 + DL2 + DL3)/3.
- E1: GPC + RS_AB.
- E2: GPC + RS_AB + RS_RB.
- E3: GPC + RS_AB + RS_RB + RS-SVM.
- E4: GPC + RS_AB + RS_RB + RS-SVM + S_D.

A + B is the sum rule applied to classifier A and classifier B, after classifier scores have been normalized to mean 0 and standard deviation 1.

In Table 5, we compare the classifier performances given in Tables 3 and 4 using the Wilcoxon Signed-Rank test. Three symbols are used in Table 5:

(i) “*L*” indicates that the method in the given row exhibits a lower performance (with *p* value < 0.10) than the method listed in the corresponding column (i.e., the classifier in that row is the “loser” compared with the column classifier).
(ii) “ND” indicates that there is no statistically significant difference between the performances of the two methods.
(iii) “*W*” indicates that the method in the given row exhibits a higher performance, with *p* value < 0.10, than the method listed in the corresponding column (i.e., the classifier in that row is the “winner” compared with the column classifier).

Analyzing the results reported in Table 5, we observe some very interesting results:

- Both SVM and RS-SVM fail to outperform any of the other state-of-the-art approaches.
- E2, E3, and E4 outperform all the other approaches.
- E4 (the fusion among all the base methods) always obtains the highest performance.
- The simple ensemble S_D obtains a performance that is comparable with all the other base methods.
- RS, which involved no parameter tuning step, outperforms SVM (implemented with LibSVM) where the parameters are optimally tuned for each dataset.

### 5.2. Experiments Results in Data Mining Datasets

The same tests reported in the previous section using the image datasets are also run for the data mining datasets. The results reported in Tables 6 and 7 clearly show the usefulness of our ensemble approach.

In these tests we obtain results that are similar to those reported in Tables 3 and 4, but there is an important difference: RS-SVM works poorly in the two datasets containing few features (HA and TR). The reason for this is simple: when only a few features are used to describe a pattern, they are likely to be uncorrelated, so an RS approach is not advised. When the datasets HA and TR are removed from consideration, RS-SVM outperforms SVM. Notice as well that in this test the ensembles outperform the base methods.

## 6. Conclusions

The aim of this paper was to compare and combine several state-of-the-art classifiers for proposing a GP ensemble that works well across a broad set of datasets (fourteen different image datasets and eleven UCI data mining datasets) with no parameter tuning. No single approach was discovered that outperformed all the other classifier systems in all the tested datasets. This finding lends support to the “no free lunch” hypothesis/metaphor that claims that “any two algorithms are equivalent when their performance is averaged across all possible problems” [40]. Nonetheless, several interesting findings are obtained when examining the classifier results across the twenty-five datasets:

- Among the different state-of-the-art methods, there is no winner.
- The GP ensembles clearly outperform the state-of-the-art methods without any complex fusion rule (the simple sum rule is used throughout the experiments). In particular, the GP ensembles outperform SVM implemented with the LibSVM toolbox, which is probably the most used classification toolbox reported in the literature.

In our opinion, a heterogeneous system based on different state-of-the-art classifiers (including classifiers that are themselves an ensemble, such as a random subspace of rotation boosting) is the most feasible way of avoiding the “curse” of the “no free lunch” metaphor.

There are many avenues for exploring GP ensembles further. A suggested list of future explorations is the following:

- Test the performance of ensembles using more complex fusion rules.
- Test systems on data mining problems where a large set of features is available.
- Expand the base methods to combine different deep learning approaches, such as an extreme learning machine [41] or convolutional neural networks [42] where the input is the whole image and not a feature vector.

## Figures and Tables

**Table 1:** Descriptive summary of the image datasets.

Dataset	Number of classes	Number of samples	Sample size	URL for download
PS	2	917	Various	http://labs.fme.aegean.gr/decision/downloads
VI	15	1500	41 × 41	http://www.cb.uu.se/~gustaf/virustexture/
CH	5	327	512 × 382	http://ome.grc.nia.nih.gov/iicbu2008/hela/index.html#cho
SM	2	2868	100 × 100	http://staff.ustc.edu.cn/~yfn/vsd.html
HI	4	2828	Various	Upon request to Loris Nanni [nanni@dei.unipd.it]
BR	2	584	Various	Upon request to Geraldo Braz Junior [ge.braz@gmail.com]
PR	2	349	Various	Upon request to Loris Nanni [nanni@dei.unipd.it]
HE	10	862	512 × 382	http://ome.grc.nia.nih.gov/iicbu2008/hela/index.html
LO	10	502	768 × 512	http://locate.imb.uq.edu.au/downloads.shtml
TR	11	553	768 × 512	http://locate.imb.uq.edu.au/downloads.shtml
PI	13	903	Various	http://imagelab.ing.unimo.it/files/bible_dataset.zip
RN	10	200	1024 × 1024	http://ome.grc.nia.nih.gov/iicbu2008/rnai/index.html
PA	13	2338	Various	http://www.cat.uab.cat/~joost/painting91.html
LE	20	1200	128 × 128	Upon request to bruno@ifsc.usp.br

**Table 2:** UCI datasets and their features: number of attributes (#A), number of samples (#S), and number of classes (#C).

Dataset	Acronym	#A	#S	#C	Brief description
BREAST	BR	9	699	2	For breast tumor diagnosis

HEART	HE	13	303	2	For detecting heart disease; the “goal” field refers to the presence of heart disease in the patient

PIMA	PI	8	768	2	For forecasting the onset of diabetes mellitus

Spam	SP	57	4601	2	For classifying E-mail as spam or nonspam

SONAR	SO	60	208	2	For discriminating between sonar signals bounced off a metal cylinder and those bounced off a rough cylindrical rock

IONOSPHERE	IO	34	351	2	For classifying radar returns from the ionosphere

Liver	LI	7	345	2	For classifying liver disorders that might arise from excessive alcohol consumption

Haberman	HA	3	306	2	A dataset that contains cases on the survival of patients who had undergone surgery for breast cancer

Vote	VO	16	435	2	For classifying Republican versus Democrat US representatives (this dataset includes votes for each member of the US House of Representatives on 16 key votes)

Australian	AU	14	690	2	For credit card applications

Transfusion	TR	5	748	2	This study adopted the donor database of Blood Transfusion Service Center; the aim is to predict whether a person donated blood in March, 2007

**Table 3:** Performance (AUC) obtained in different image datasets using LTP as texture descriptor.

LTP	Datasets (AUC)														Av	RA
	PS	VI	CH	SM	HI	BR	PR	HE	LO	TR	PI	RN	PA	LE		
SVM	0.9144	0.9349	**1.000**	0.9975	0.9156	0.9692	0.8968	0.9814	0.9949	0.9926	0.9286	0.9696	0.8903	0.9792	0.9546	7.0
RS-SVM	0.9071	0.9352	**1.000**	**0.9976**	0.9195	0.9763	0.9030	0.9826	0.9950	0.9924	0.9316	0.9713	0.8944	0.9807	0.9562	5.9
GPC	0.9086	0.9131	0.9997	0.9971	0.9198	0.9789	0.8865	0.9816	0.9964	0.9930	0.9090	0.9769	0.8968	0.9752	0.9523	8.1
RS_AB	0.9121	0.9254	0.9998	0.9974	0.8924	0.9810	0.9079	0.9813	0.9965	0.9953	0.9242	0.9771	0.8959	0.9738	0.9543	7.0
RS_RB	0.9110	0.9293	0.9999	0.9953	0.9136	0.9739	0.8886	0.9806	0.9969	0.9955	0.9178	0.9900	0.8940	0.9738	0.9543	7.6
DL1	0.8927	0.9173	0.9999	0.9952	0.9072	0.9811	0.8486	0.9801	0.9962	0.9965	0.9147	0.9837	0.8865	**0.9811**	0.9486	8.7
DL2	0.8965	0.9220	0.9998	0.9956	0.8945	0.9815	0.8780	0.9806	0.9956	0.9959	0.9061	0.9878	0.8869	0.7900	0.9365	9.4
DL3	0.7802	0.9239	0.9999	0.9963	0.9082	0.9815	0.8779	0.9812	0.9958	0.9962	0.9014	0.9916	0.8895	0.9525	0.9412	8.5
S_D	0.8985	0.9244	1.000	0.9958	0.9143	**0.9835**	0.8783	0.9818	0.9958	0.9966	0.9151	**0.9916**	0.8960	0.9806	0.9537	6.0
E1	0.9130	0.9196	0.9997	0.9974	0.9162	0.9812	0.8999	0.9816	0.9962	0.9945	0.9191	0.9798	0.8983	0.9748	0.9551	7.2
E2	**0.9165**	0.9337	0.9998	0.9973	0.9184	0.9809	0.9007	0.9837	0.9969	0.9960	0.9238	0.9884	0.9030	0.9768	0.9583	5.1
E3	**0.9165**	0.9361	**1.000**	0.9975	**0.9229**	0.9816	**0.9090**	0.9843	0.9970	0.9968	0.9313	0.9835	0.9080	0.9796	0.9603	2.8
E4	0.9164	**0.9372**	**1.000**	0.9975	0.9235	0.9824	0.9059	**0.9851**	**0.9973**	**0.9971**	**0.9318**	0.9864	**0.9093**	0.9808	**0.9608**	**2.2**

**Table 4:** Performance obtained on the different image datasets using LPQ as texture descriptor.

LPQ	Datasets (AUC)														Av	RA
	PS	VI	CH	SM	HI	BR	PR	HE	LO	TR	PI	RN	PA	LE		
SVM	0.9039	**0.9492**	0.9999	0.9986	0.9138	0.9565	0.8618	0.9757	0.9764	0.9767	0.9071	0.9532	0.8834	**0.9897**	0.9461	8.4
RS- SVM	0.8951	0.9485	0.9999	0.9988	0.9251	0.9568	0.8727	0.9786	0.9809	0.9817	0.9128	0.9531	0.8854	0.9891	0.9485	7.5
GPC	0.9020	0.9282	0.9991	0.9985	0.9199	0.9720	0.8883	0.9793	0.9891	**0.9934**	0.9073	0.9439	0.8867	0.9782	0.9490	7.4
RS_AB	0.9013	0.9417	0.9998	0.9989	0.8783	0.9671	0.8843	0.9781	0.9868	0.9907	0.9255	0.9478	0.8777	0.9826	0.9472	7.9
RS_RB	0.8994	0.9393	0.9992	0.9978	0.9120	0.9711	0.8999	0.9741	0.9800	0.9889	0.9116	0.9562	0.8806	0.9799	0.9493	8.6
DL1	0.8701	0.9382	0.9994	0.9982	0.9083	0.9684	0.8758	0.9815	0.9847	0.9873	0.9110	0.9537	0.8858	0.9819	0.9460	9.0
DL2	0.8081	0.9379	0.9989	0.9979	0.9025	0.9682	0.8745	0.9813	0.9851	0.9852	0.9033	0.9550	0.8783	0.9853	0.9401	10.2
DL3	0.8717	0.9401	0.9990	0.9983	0.9097	0.9647	0.8694	0.9813	0.9861	0.9854	0.9038	**0.9666**	0.8785	0.9833	0.9456	9.3
S_D	0.8864	0.9415	0.9997	0.9982	0.9165	0.9687	0.8807	**0.9830**	0.9871	0.9885	0.9118	0.9594	0.8894	0.9848	0.9497	6.3
E1	0.9045	0.9345	0.9994	0.9989	0.9137	0.9726	0.8884	0.9794	0.9899	0.9931	0.9202	0.9469	0.8860	0.9807	0.9506	6.3
E2	0.9065	0.9441	0.9995	**0.9991**	0.9168	**0.9742**	0.8942	0.9793	0.9883	0.9932	0.9219	0.9574	0.8910	0.9834	0.9535	4.2
E3	**0.9103**	0.9467	0.9999	0.9990	0.9238	0.9716	0.8968	0.9805	0.9891	0.9927	0.9228	0.9581	0.8981	0.9867	0.9554	3.3
E4	0.9097	**0.9492**	**1.000**	0.9990	**0.9258**	0.9714	**0.8978**	0.9825	**0.9902**	0.9926	**0.9235**	0.9635	**0.8998**	0.9870	**0.9566**	**2.2**

**Table 5:** Comparisons between all the pairs of tested methods.

	SVM	RS-SVM	GPC	RS_AB	RS_RB	DL1	DL2	DL3	S_D	E1	E2	E3	E4
SVM	—	L	ND	L	ND	ND	ND	ND	ND	L	L	L	L
RS-SVM	—	—	ND	L	ND	ND	ND	ND	ND	L	L	L	L
GPC	—	—	—	ND	ND	ND	W	W	ND	L	L	L	L
RS_AB	—	—	—	—	ND	W	W	W	ND	ND	L	L	L
RS_RB	—	—	—	—	—	W	W	ND	ND	L	L	L	L
DL1	—	—	—	—	—	—	ND	ND	L	L	L	L	L
DL2	—	—	—	—	—	—	—	ND	L	L	L	L	L
DL3	—	—	—	—	—	—	—	—	ND	L	L	L	L
S_D	—	—	—	—	—	—	—	—	—	ND	L	L	L
E1	—	—	—	—	—	—	—	—	—	—	L	L	L
E2	—	—	—	—	—	—	—	—	—	—	—	L	L
E3	—	—	—	—	—	—	—	—	—	—	—	—	L

**Table 6:** Performance on the different data mining datasets.

	Datasets (AUC)											Av	RA
	BR	HE	PI	SP	SO	IO	LI	HA	VO	AU	TR		
SVM	0.9941	0.8809	0.824	0.9708	0.9517	0.9814	0.7558	**0.7012**	0.9855	0.9164	0.714	0.8796	7.3077
RS-SVM	0.9931	0.9076	0.8221	0.9771	**0.9591**	0.9795	0.7411	0.6399	0.9853	0.9221	0.6931	0.8745	8.6923
GPC	0.9924	0.9024	0.827	0.979	0.9409	0.9713	0.729	0.6804	0.9882	0.9267	0.7295	0.8788	8.000
RS_AB	0.991	0.9101	0.8229	**0.988**	0.9371	0.9788	0.7581	0.6727	0.9887	0.9313	0.735	0.8831	7.000
RS_RB	0.9925	**0.9169**	0.8208	0.9873	0.9334	**0.9851**	0.7664	0.6071	0.9884	0.9326	0.674	0.8731	7.3846
DL1	**0.9943**	0.8852	0.8252	0.966	0.8794	0.9222	0.7541	0.6751	0.9795	0.9155	0.7338	0.8664	8.7692
DL2	0.9941	0.8754	0.8149	0.9691	0.8789	0.9242	0.7478	0.6679	0.9808	0.9088	0.7318	0.8631	10.3077
DL3	**0.9943**	0.8941	0.8193	0.9684	0.8501	0.9022	0.6966	0.6537	0.9787	0.9154	0.7351	0.8553	10.2308
S_D	0.9942	0.883	0.8238	0.9683	0.8781	0.9297	0.751	0.6772	0.9813	0.9186	0.7357	0.8674	8.6154
E1	0.992	0.9096	0.8277	0.9856	0.9426	0.9772	0.7532	0.6868	**0.9896**	0.9331	**0.7372**	0.885	6.000
E2	0.9924	0.9124	0.8285	**0.988**	0.9426	0.9817	**0.7727**	0.6724	0.9897	**0.935**	0.7257	0.8856	5.3846
E3	0.9933	0.9141	0.8288	0.9873	0.9508	0.9819	0.7723	0.6726	0.989	0.9343	0.7258	**0.8864**	**5.0769**
E4	0.9934	0.9113	**0.8294**	0.9862	0.942	0.9805	0.7717	0.6794	0.9895	0.9339	0.7297	0.8861	5.2308

**Table 7:** Comparisons between all the pairs of methods tested in Table 6.

	SVM	RS-SVM	GPC	RS_AB	RS_RB	DL1	DL2	DL3	S_D	E1	E2	E3	E4
SVM	—	ND	ND	ND	ND	W	W	W	W	ND	L	L	L
RS-SVM	—	—	ND	ND	W	ND	ND	W	ND	L	L	L	L
GPC	—	—	—	L	ND	W	W	W	W	L	L	L	L
RS_AB	—	—	—	—	ND	W	W	W	W	ND	L	L	L
RS_RB	—	—	—	—	—	ND	ND	W	ND	ND	L	L	L
DL1	—	—	—	—	—	—	W	W	ND	L	L	L	L
DL2	—	—	—	—	—	—	—	W	ND	L	L	L	L
DL3	—	—	—	—	—	—	—	—	L	L	L	L	L
S_D	—	—	—	—	—	—	—	—	—	L	L	L	L
E1	—	—	—	—	—	—	—	—	—	—	ND	ND	ND
E2	—	—	—	—	—	—	—	—	—	—	—	ND	ND
E3	—	—	—	—	—	—	—	—	—	—	—	—	ND
